# Increase in frequencies of circulating Th-17 cells in HIV-1 infected patients with poor CD4+ T-cell reconstitution on effective HAART

**DOI:** 10.1186/1471-2334-14-S2-P65

**Published:** 2014-05-23

**Authors:** D Asthana, D Gracia, R Valiathan

**Affiliations:** 1 University of Miami, Miami, USA

## Introduction

Although circulating Th-17 (cTh-17) cells are related to inflammatory diseases, its role in HIV immunopathogenesis is not well known. We investigated the association of cTh-17 cells with immune activation (IA), immune exhaustion (IE) and regulatory T-cells (T-regs) in HAART treated virologically controlled (<40 HIV RNA copies/ml) HIV-1 infected patients with poor CD4 T-cell recovery (discordant).

## Materials and methods

Discordant patients (n=20) with mean absolute CD4 numbers (absCD4: 273±29) were compared with virologically controlled patients on HAART (concordant, n=20) with optimal CD4 T-cell recovery (absCD4:852±51) and 10 healthy controls (HCs). cTh17 cells were measured in PBMCs, as frequencies of CD4+IL-17+ cells, after PMA+Ionomicin stimulation by flow cytometry, and correlated with markers of IA (HLA-DR+CD38+) and IE (Tim-3, PD-1 and CTLA-4) of CD4 and CD8 T-cells and frequencies of T-regs (CD4+CD25brightFoxP3+). Plasma levels of lipopolysaccharide (LPS) and soluble-CD14 (sCD14) were measured to determine gut microbial translocation (MT). Results obtained for discordant patients were compared with concordant patients and HCs.

## Results

Frequencies of cTh-17 cells were significantly higher in discordant patients than concordant patients (P < 0.0001) and HCs (P = 0.014). cTh-17 cells frequency correlated inversely with CD4 T-cell percentages and absolute count and directly with CD4-IA and T-reg frequencies. Discordant patients showed higher CD4-IA (HLA-DR+CD38+) than concordant patients which directly correlated with MT. CD4 T-cells of discordant patients showed higher expression of multiple IE markers (PD1+Tim3+) that correlated with IA. Although naive CD4+ T-cells were lower, effector memory cells were higher in discordant patients.

## Conclusions

Persistent CD4 T-cell IA and inflammation in discordant patients may favor the differentiation of activated CD4 T-cells towards cTh-17 phenotype. cTh-17 cell frequency could be an indicator of ongoing IA and inflammation in discordant patients. Analysis of the nature and function of cellular subsets associated with IA and immune regulation would assist in understanding HIV immunopathogenesis and developing strategies to target disease progression.

